# Selenite benefits embryonic stem cells therapy in the animal models of Parkinson’s disease through inhibiting inflammation

**DOI:** 10.1186/1750-1326-7-S1-L25

**Published:** 2012-02-07

**Authors:** Lipeng Tian, Shi Zhang, Liang Xu, Wen Li, Ying Wang, Wei Chen, Jianqing Ding, Shengdi Chen

**Affiliations:** 1 Department of Neurology & Institute of Neurology, Ruijin Hospital affiliated to Shanghai Jiao Tong University School of Medicine, Shanghai 200025, China

## 

Embryonic stem cells (ESCs) transplantation is a potential therapeutic approach for Parkinson’s disease. However, the key problems the therapy is facing are the efficiency of differentiation into dopaminergic (DA) neurons and the low survival of the transplanted DA neurons. In the present study, mouse ESC were effectively differentiated into DA neurons by serum free method and were transplanted into 6-OHDA lesioned striatum of PD rats. We found reduced viability of DA neurons after graft, being accompanied by activated microglia and high levels of TNF-α and iNOS. This suggested that inflammation might be an underlying mechanism for decreased cells viability. In the following in vitro assay, selenite, the source of essential micronutrient selenium, was tested to inhibit inflammatory activation of BV2 microglia cells. Furthermore, the anti-inflammatory effects of selenite in animals after cells transplantation were investigated. In PD rats treated by selenite, microglia activation after transplantation was inhibited in the graft niche, and the levels of TNF-α and iNOS were effectively abated nearly by 30% and 50%. The viability of implanted DA neurons was also remarkably improved after selenite treatment, with favored behavior recovery of PD rats. Therefore, selenite might benefit embryonic stem cells therapy in Parkinson’s disease through inhibiting inflammation.

